# Application of Various Types of Liposomes in Drug Delivery Systems

**DOI:** 10.15171/apb.2017.002

**Published:** 2017-04-13

**Authors:** Mehran Alavi, Naser Karimi, Mohsen Safaei

**Affiliations:** Department of nanobiotecnology, 67149 Baghabrisham, Razi University, Kermanshah, Iran.

**Keywords:** Liposomes, Hydrophobic drugs, Phospholipid, Drug delivery system

## Abstract

Liposomes, due to their various forms, require further exploration. These structures can deliver both hydrophilic and hydrophobic drugs for cancer, antibacterial, antifungal, immunomodulation, diagnostics, ophtalmica, vaccines, enzymes and genetic elements. Preparation of liposomes results in different properties for these systems. In addition, based on preparation methods, liposomes types can be unilamellar, multilamellar and giant unilamellar; however, there are many factors and difficulties that affect the development of liposome drug delivery structure. In the present review, we discuss some problems that impact drug delivery by liposomes. In addition, we discuss a new generation of liposomes, which is utilized for decreasing the limitation of the conventional liposomes.

## Introduction

In the late nineteenth century, German bacteriologist Paul Ehrlich, used the term “magic bullet,” which means chemical carriers that have the property of selectivity in killing abnormal cells without any effect on the normal ones.^1^ In order to improve this specificity through drug delivery systems, there are a variety of different approaches, which are based on a number of physical and bio-chemical principles.^2^

Liposomes, as carrier systems, have been explored more than any than other system as a result of their various forms. Phospholipid bilayers membranes can generate sphere structure with internal hydrophilic compartment through introducing phospholipids in water solution; these structures are called liposomes. About four decades ago, Bangham and co-workers defined liposomes as vesicles with small size and spherical shapes that can be generated from phospholipids, cholesterols, non-toxic surfactants and even membrane protein. Investigations of this group resulted in regarding liposomes as delivery systems, which are characterized by carrying a variety of compounds in the core section.^3,4^ These structures can encapsulate and deliver both hydrophilic and hydrophobic substances afflictively.

## Raw Materials in Liposome Preparation

**Figure 1:**
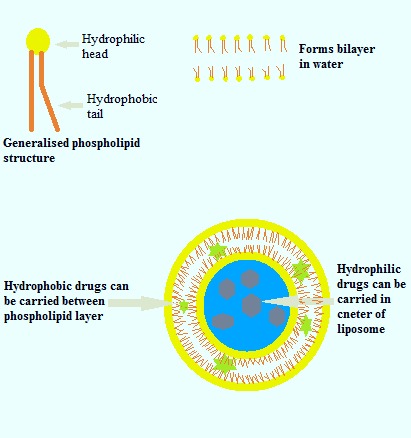
Schematic Drawing of Liposome Structure and Its Compartments

Lipids are amphipatic molecules with water-friendly and water-hating parts (Figure 1). Liposomes are consisted of single or multiple lipid bilayers formed by hydrophilic and hydrophobic interactions with the aqueous phase. The hydrophobic parts (tails) of liposomes are repelled by water molecules resulting in liposome self-assembly.^5^ In addition, Phosphatidylcholine (PC) and Dipalmitoyl PC can be used for liposome generation, respectively. Two important advantages of liposomes, in drug delivery of living organisms, are biocompatibility and biodegradability, which are due to lipid characteristics.^6^ Different types of lipids and amphiphiles can act as liposomes (Table 1); furthermore, polymers can be used for the synthesis of polymerosomes as new drug/gene carriers.

## Liposomes Preparation

There are several approaches for preparation of liposomes, which include the use of mechanical procedures, organic solvents, or through the removal of detergent from phospholipid/detergent micelle mixtures. In liposome preparation, types and amounts of phospholipid, the ionic and charge properties of aqueous medium, as well as time hydrations, are important factors that determine the final liposome structure.^7^

**Table 1:** Various Lipids and Amphiphiles that are Used as Liposome Raw Materials

**Natural phospholipids**	**Synthetic phospholipids**	**Unsaturated**	**Sphingolipids**	**Glycosphingolipids**	**Steroids**	**Polymeric material**	**Charge-inducing lipids**
Phosphotidylcholine	1,2- Dilauroyl-sn-Glycero-3-Phosphocoline (DLPC)	1-Stearoyl-2-Linoleoyl-sn-Glycero-3-[Phospho-L-Serine] (Sodium Salt)	Shingomyellin	Gangliosides	Cholesterol	Lipids conjugated to diene, methacrylate & thiol group	Diotadecyldimethyl ammonium bromide/chloride (DODAB/C); Dioleoyl trimethylammonium propane (DOTAP)
Phosphotidylserine	1,2-Dioleoyl-sn-Glycero-3-[Phospho-L-Serine] (Sodium Salt) (DOPS)	Dioleaylphosphotidylcholine					
Phosphotidylethanolamine	Dipalmitoylphosphotidylcholine						
phosphotidylinositol	Distearoylphosphotidylcholine						
	Dipalmitoylphosphotidylseine						
	Dipalmitoylphosphotidylglycerol						
	1,2-Dilauroyl-sn-Glycero-3-Phosphocholine (DLPC)						

### Multilamellar vesicles preparation 

Production of multilamellar vesicles is the simplest method in all liposome preparations. In this method, stages of liposome generation are used as organic solvent for dissolving of lipid and drying of the resulted mixture. Combination of lipids such as egg lecithin, cholesterol and phosphatidyl glycerol in a molar ratio of 0.9:1.0:0.1 are used respectively. Chloroform or a mixture of chloroform and methanol in a typical ratio of 2:1 are used respectively. Firstly, each lipid component is dissolved in the organic solvent separately, followed by mixing in the suitable proportion with the other solubilized lipids to ensure and uniform distribution of the lipids in mixture. Afterwards, nitrogen stream is used to generate a film from the mixture in test tube. Also, in order to remove any last traces of organic solvent, the film of lipid is allowed to dry completely in an evacuated chamber for a minimum of 4-6 hours.^8^

### Unilamellar vesicles preparation 

The unilamellar vesicle is the most popular type of liposomes. Its liposome structure allows for an even distribution of trapped agents within a single internal aqueous compartment. There are several methods for preparation of these structures including ultrasonication, extrusion through polycarbonate filters, freeze-thawing, ethanol injection, detergent method and preparation of sterile large unilamellar vesicles. Bhatia et al (2015) used mixture of different small unilamellar vesicles (SUVs) populations for obtain ternary GUV with uniform property.^9^

### Giant Unilamellar Liposomes Preparation 

There are many methods in the preparation of giant liposomes based on utilizing only distilled water, non-electrolyte or zwitterions. There is an increase in attraction between membranes caused by the presence of ions imparting a net charge, and thereby inhibiting the separation of the membrane sheets during the rehydration and swelling process. Recently, researchers have demonstrated preparation of giant liposomes, using physiological strength buffers (Table 2). There are several methods for preparation of these systems including electroformation, giant liposomes prepared in rapid preparation, using physiological buffer for preparation of giant unilamellar liposomes and osmotic shock technique.^10^ Also, Karamdad and coworkers (2015) used new method of a microfluidic for GUV preparation and mechanical characterization.^11^

**Table 2:** Advantages and Disadvantages of Giant Unilamellar Liposomes Preparation Methods.

	**Advantages**	**Disadvantages**
Electroformation^12^	Production of immobilized giant liposoems	To apply in low ionic strength buffers (equals to or less than 2 V)
Rapid preparation of giant liposomes^13^	Fast single – step procedure	Buffer with ionic low strength (a maximum of 50mM)
Giant unilamellar prepared in physiological buffer^14^	Using various physiological salt solutions, such as 100mM KCl plus 1mM CaCl_2_	Time consuming procedure

## Loading of Drugs by Liposomes

### Encapsulation of Hydrophilic Drugs

Encapsulation of‏ hydrophilic drugs results in hydration of lipids hydrophilic drugs mixture. Through such a method, drugs can enter the liposome core and other materials remain in outside part of the liposome. Remained materials will remove drug entrapment in liposome. In order to purify these two parts (drugs and remained outside materials), gel filtration column chromatography and dialysis are used. In addition, dehydration and rehydration method may be applied for high encapsulation of the DNA and proteins.^15^

### Encapsulation of Hydrophobic Drugs

The phospholipid bilayer of liposomes is a region of hydrophobic drug encapsulation. By entrapment of this type of drugs (such as verteporfin (Visudyne)), movement of drug will be be decreased towards the outer aqueous and inner parts of liposomes. These drugs are encapsulated through solubilizing of drug in the organic solvent and phospholipids. Region of drug entrapement in liposome is the hydrophobic part of liposome. Afterwards, it is possible to use laser light for activation of drug due to the treatment of wet macular degeneration.^16^

## Liposomes and Clinical Applications

One of the most efficient nano-systems, with several approved formulations for diseases treatment, are Liposomes. These systems have unique properties such as smaller size, biodegradability, biocompatibility, hydrophobic and hydrophilic character, low toxicity and immunogenicity that results in significant efficiency on cancer therapy.^17^

### Archaeosomes

*Archaeobacteria* is a Domain of prokaryotes that is different from *Eukarya* and *Bacteria* domains.^18^ Solvent extraction is main method for obtaining total lipids of archaeobacteria. As illustrated about, in this method, 5% of the total lipids of the cell dry weight. The significant core lipid structures, known to be present in archaeobacteria, are Archaeol (As), Macrocyclic Archaeol (A_m_), 3´-hydroxy archaeol (A_OH_), Caldarchaeol (C_s_), Nanitol-caldarchaeol (C_n_) and Cyclopentane-caldarchaeol (C_p_). Although, some of archaeobacteria have various amounts of hydroxyl archaeols (hydroxyl diethers), their exact function remains unknown. Some types of this bacteria such as *Halobacterium cutirubrum* and *Methanosarcina mazei* contain only archaeol lipids and Thermoplasma acidophilum from thermophilic type consist of caldarchaeol lipids (90% of the total polar lipids). Table 3 shows the specific core lipids types in some of the archaeobacteria that are used in various studies on membranes and in vesicles formation.

**Table 3:** The Relative Abundance (%) of the Specific Core Lipids in Archaobacteris Used in Vesicle Formation^a^

**Archaeobacteria**	**Standard archaeol**	**Standard caldarchaeol**	**Hydroxyarchaeol**
*Halobacterium cutirubrum*	100	—	—
*Methanococcus voltae*	>90	—	˂10
*Methanosphaera stadtmanae*	79	13	8
*Methanococcus jannaschii* (50 ˚C)	60	21	—
*Methanobrevibacter smithii*	58	40	2
*Methanospirillum hungatei*	50	50	—
*Methanosarcina mazei*	43	—	57
*Mthanobacterium espanolae*	35	65	—
*Methanococcus jannaschii* (65 °C)	15	42	—
*Thermoplasma acidophilum*	˂9	90	>1
*Sulfolobus acidocaldarius*	˂9	90	>1

^a^ Data summarized from Patel and Sprott (1999).^19^

Archeobacteria membranes have diether and/or tetraether linkages that are used for generation of lipid layers of archeosomes.^20^ An advantage of archeobacterial lipids in comparison to conventional liposomes is higher stability in harsh condition.^21^ Sonication, extrusion after hydration of lipid thin films, as well as detergent dialysis can be utilized for archaeosomes preparation at wide range of temperatures, which include physiological to lower temperatures. The higher efficiency of archeosomes in drug/gene delivery is resulted from the biocompatibility and higher stability of these systems.^14^

### Niosomes

**Figure 2:**
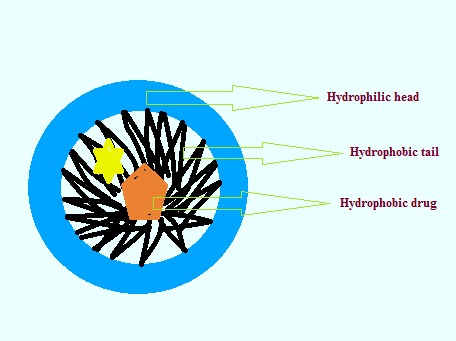
Niosome structure.

Preparation of liposomes by nonionic surfactants such as alkyl ethers or alkyl esters results in niosome structures. An advantage of niosomes is storage and handling with biodegradable, biocompatible and non-immunogenic properties without any specific conditional requirements. The oral bioavailability of drugs with low absorption efficiency can be increased by these delivery systems; in addition, they have suitable impact on the clearance of the drug from reticulocyte endothelial system (RES) and lead to therapeutic effect of drugs^22^ (Figure 2).

The suitable formulations of encapsulated enoxacin by liposomes and niosomes were studied for improvement of dermal delivery. Also, the optimized formulations demonstrated a large amount of enoxacin in the skin. This study showed a high stability of noisome compared to liposomes after 48 h incubation. Yongmei and coworkers (2002) reported an enhanced method for encapsulation of colchicine by niosomes; in their work, the encapsulation capacity was high and the side effects were low for these systems.^23^

### Novasomes 

**Figure 3:**
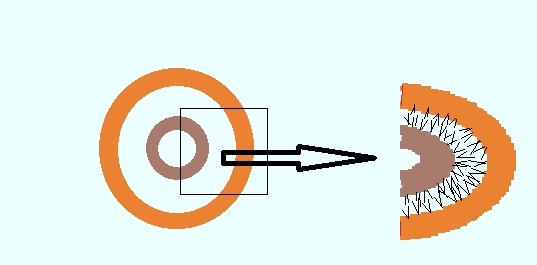
Novasome vesicle.

Novasomes are produced by mixture of polyoxyethylene fatty acids (as monoester), free fatty acids and cholesterol (Figure 3). The diameter range of novasomes is 0.1 up to 1 micron. These systems have 2-7 bilayers and a large amphipathic core with 80-85% of drug loading. In addition, the Charge of novasomes surfaces may be neutral, positive or negative.^24^

Novasomes can capsulate both hydrophilic and hydrophobic drug molecules, and can be encapsulated by novasomes. Moreover, it is possible for entering drugs in bilayers and therefore prevent the incompatibility of drugs in surface charge properties. These systems can deliver a high amount of ingredients specifically in the cosmetic.^25^ Based on novasomes, various vaccines have been patented; also, there are other vaccines against bacterial and viral infections such as small pox vaccine. Novasomes can fuse with enveloped virus and denature virus shortly after fusion.^26^

### Cryptosomes

Mononuclear phagocytic system (MPS) clears conventional liposomes from RES. In order to limit such a drawback, polyethylene glycol (PEG) derivatives are suitable choices. This type of liposome modification is called stealth liposome or cryptosome. Cryptosomes decrease contact and uptake by MPS, through high amount of PEG on their surface. The result of such a characteristic is the augmentation in the time of circulation. Also, active targeting of cryptosomes get possible by application of various ligands. Poloxamer have nonionic triblock copolymers structure of a central hydrophobic chain of polyoxypropylene, surrounded by two hydrophilic chains of polyoxyethylene. These structures are used in cryptosomes preparation, and some of which can be incorporate into the bilayers and production of micelles.^22^

### Emulsomes

**Figure 4:**
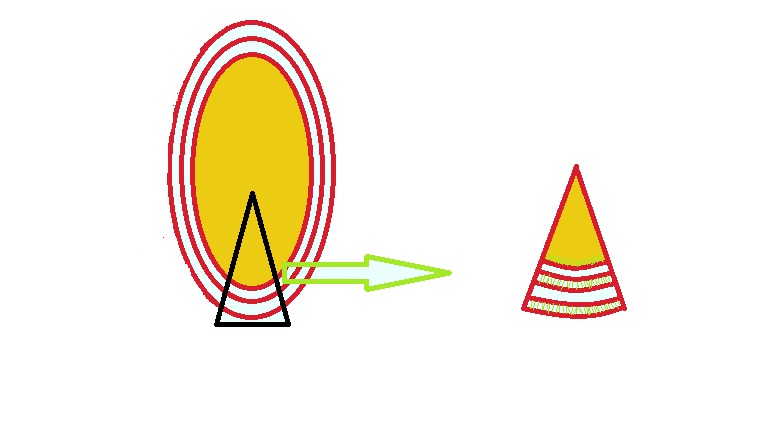
Structure of emulsome.

Emulsomes have phospholipid bilayer with a solid fat core (Figure 4). These structures have properties of emulsion and liposomes. Solid fat core is enclosed by ones or more phospholipid bilayers. Internal core of emulsomes is different and affect the hydrophobic drug loading. In order to produce emulsomes of smaller size, the drug loading is followed by sonication.^27^ Many types of stabilizers, such as soya lecithin or cholesterol can be utilized for the improvement of oil-in-water emulsion formation.

Emulsomes are more stable than other vesicular systems because of their micro or nano-scale size. Consequently, they have higher efficiency than conventional liposome in drug releasing and entrapment. In this case, the stability of emulsomes were reported up to 24 hours in comparison to 6 hours for liposomes.^28^ Furthermore, the entrapment efficiency of emulsomes for silybin in secondary metabolite can escalate to 80%.^29^ After intravenous injection of nanoemulsomes, there is absorption passively from RES through macrophages of liver. This function related to colloidal nature of this system and is effective for lever decease treatment such as cirrhosis.^29^

### Vesosomes 

Vesosomes is a multi-compartmental structure of lipid vesicles, derived from liposomes, which are potentially powerful models used to deliver drugs. These structures include membrane-bound vesicles, which encapsulate drugs in their core part vesicles.^30^ The function of external bilayers is the protection of the drug form degradation by enzymes and other defensive elements of human body.^31^ Considerable advantages of vesosomes are simple preparation and multiple drugs loading that are important in cancer treatment with resistance to special drugs.

## Conclusion

Liposomes are significant candidates for the improvement of drug delivery systems. Recent studies illustrate the great potential for the widespread adoption of liposomes in cancer treatment.^32^ These structures have major characteristics including low toxicity, biocompatibility, lower clearance rates, the ability to target cancer tissues and controlled release of drugs. Liposomes provide a number of advantages than conventional chemotherapy through free drug treatment, as evidenced by the approval of Doxil.‏ Depending on the size, lamellar number and form and formulation of constitutes, there are several types of liposomes. Clinical usages of these systems cover diagnostic, therapeutic, vaccine improvement. Drug and gene delivery are two therapeutic aspects, in which liposomes can be effective due to their specific properties. Many diseases were investigated regarding the involvement liposomes in treatment and some of results were satisfactory. Among these diseases, cancer is the most prominent. In this regard, both imaging and chemotherapy were surveyed by these structures. These studies lead to various formulations of liposomes in several clinical phases on the market (Table 4). An important issue is the use of different kinds of liposomal formulations in clinical trials, which is typically more difficult than conventional liposomal types. In this case, none of the formulations provide a complete system, and in spite of their efficiency, each one has its own deficiency. Therefore, in order to expand the desirable aspects of drug delivery systems in clinical trials, more investigations is necessary.

**Table 4:** Liposomal formulations on the market.

**Company**	**Product**	**Status**	**References**
Liposome Co., Princeton, NJ, USA	DC99: liposomal doxorubicin Ventus: liposomal PGE_1_	Phase III Phase III not successful	^33^ ^34^
Asta Medica, Frankfurt, Germany Aronex, The Woodlands, TX, USA	Topical anticancer cream Nyotran: liposomal nystatin Liposomal annamycin Atragen: liposomal retinoic acid	On German market Phase III Phase II Phase II	^34^ ^35^ ^34^
Inex, Vancouver, BC, Canada Swiss Serum Institute, Bern, Switzerland	Liposomal vincristine Epaxal: hepatitis-A vaccine Trivalent influenza vaccine Hepatitis-A and B vaccine Diphtheria, tetanus and hepatitis-A vaccines Diphtheria, tetanus, influenza and hepatitis-A vaccine	Phase I On Swiss market since 1994 Phase III Phase I Phase I Phase I	^36^
NeXstar, Boulder, CO, USA	Spy 07: cisplatin in stealth liposomes Ambisome: amphotericin B in liposomes DaunoXome: daunorubicin in liposomes Mikasome: liposomal amikacin	Phase I On the market since 1990 (Europe) and 1997 (USA) On the market since 1996 (USA and Europe) Phase I	^37^ ^38^ ^39^ ^40^
Novavax, Rockville, MD, USA	*Escherichia Coli* vaccine in synthetic liposomes *Shigella flexneri* vaccine in synth. liposomes	Phase I Phase I	^34^
IGI, Vineland, NJ, USA (veterinary)	Newcastle-disease vaccine (chicken) Avian-reovirus vaccine	On the market On the market	^41^
Biozone Labs, Pittsburgh, CA, USA	ELA-Max: liposomal lidocaine	On the US market since 1998	^42^
Sequus, Menlo Park, CA, USA	Doxil: doxorubicin in stealth liposomes	On the market since 1995 (USA) and 1996 (Europe)	^43^

## Ethical Issues

Not applicable.

## Conflict of Interest

The authors declare no conflict of interests.
